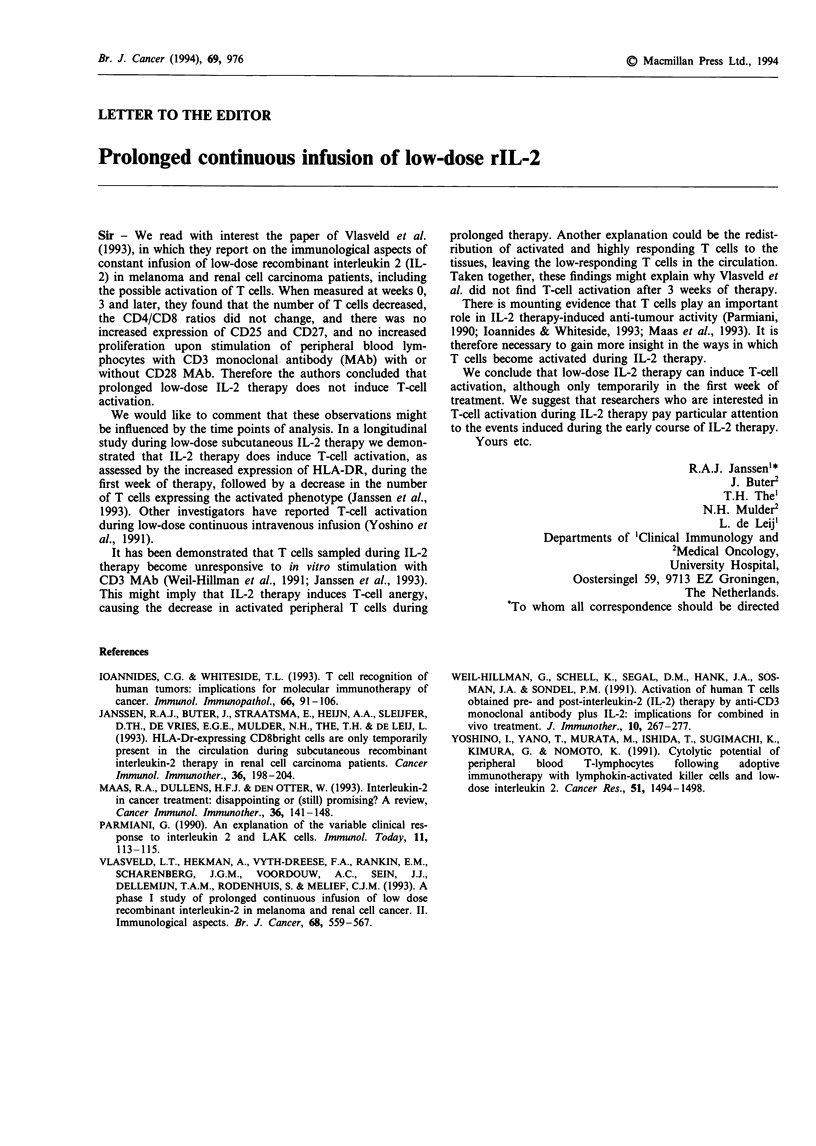# Prolonged continuous infusion of low-dose rIL-2.

**DOI:** 10.1038/bjc.1994.189

**Published:** 1994-05

**Authors:** R. A. Janssen, J. Buter, T. H. The, N. H. Mulder, L. de Leij


*Br. J. Cancer* (1994), **69**, 976 © Macmillan Press Ltd., 1994

### LETTER TO THE EDITOR

### Prolonged continuous infusion of low-dose rIL-2

**Sir** – We read with interest the paper of Vlasveld *et al.* (1993), in which they report on the immunological aspects of constant infusion of low-dose recombinant interleukin 2 (IL-2) in melanoma and renal cell carcinoma patients, including the possible activation of T cells. When measured at weeks 0, 3 and later, they found that the number of T cells decreased, the CD4/CD8 ratios did not change, and there was no increased expression of CD25 and CD27, and no increased proliferation upon stimulation of peripheral blood lymphocytes with CD3 monoclonal antibody (MAb) with or without CD28 MAb. Therefore the authors concluded that prolonged low-dose IL-2 therapy does not induce T-cell activation.

We would like to comment that these observations might be influenced by the time points of analysis. In a longitudinal study during low-dose subcutaneous IL-2 therapy we demonstrated that IL-2 therapy does induce T-cell activation, as assessed by the increased expression of HLA-DR, during the first week of therapy, followed by a decrease in the number of T cells expressing the activated phenotype (Janssen *et al.*, 1993). Other investigators have reported T-cell activation during low-dose continuous intravenous infusion (Yoshino *et al.*, 1991).

It has been demonstrated that T cells sampled during IL-2 therapy become unresponsive to *in vitro* stimulation with CD3 MAb (Weil-Hillman *et al.*, 1991; Janssen *et al.*, 1993). This might imply that IL-2 therapy induces T-cell anergy, causing the decrease in activated peripheral T cells during prolonged therapy. Another explanation could be the redistribution of activated and highly responding T cells to the tissues, leaving the low-responding T cells in the circulation. Taken together, these findings might explain why Vlasveld *et al.* did not find T-cell activation after 3 weeks of therapy.

There is mounting evidence that T cells play an important role in IL-2 therapy-induced anti-tumour activity (Parmiani, 1990; Ioannides & Whiteside, 1993; Maas *et al.*, 1993). It is therefore necessary to gain more insight in the ways in which T cells become activated during IL-2 therapy.

We conclude that low-dose IL-2 therapy can induce T-cell activation, although only temporarily in the first week of treatment. We suggest that researchers who are interested in T-cell activation during IL-2 therapy pay particular attention to the events induced during the early course of IL-2 therapy.

Yours etc.

R.A.J. Janssen[1]*
J. Buter[2]
T.H. The[1]
N.H. Mulder[2]
L. de Leij[1]
Departments of [1]Clinical Immunology and
[2]Medical Oncology,
University Hospital,
Oostersingel 59, 9713 EZ Groningen,
The Netherlands.
*To whom all correspondence should be directed

### References

IOANNIDES, C.G. & WHITESIDE, T.L. (1993). T cell recognition of human tumors: implications for molecular immunotherapy of cancer. *Immunol. Immunopathol.*, **66**, 91–106.

JANSSEN, R.A.J., BUTER, J., STRAATSMA, E., HEIJN, A.A., SLEIJFER, D.TH., DE VRIES, E.G.E., MULDER, N.H., THE, T.H. & DE LEIJ, L. (1993). HLA-Dr-expressing CD8bright cells are only temporarily present in the circulation during subcutaneous recombinant interleukin-2 therapy in renal cell carcinoma patients. *Cancer Immunol. Immunother.*, **36**, 198–204.

MAAS, R.A., DULLENS, H.F.J. & DEN OTTER, W. (1993). Interleukin-2 in cancer treatment: disappointing or (still) promising? A review, *Cancer Immunol. Immunother.*, **36**, 141–148.

PARMIANI, G. (1990). An explanation of the variable clinical response to interleukin 2 and LAK cells. *Immunol. Today*, **11**, 113–115.

VLASVELD, L.T., HEKMAN, A., VYTH-DREESE, F.A., RANKIN, E.M., SCHARENBERG, J.G.M., VOORDOUW, A.C., SEIN, J.J., DELLEMIJN, T.A.M., RODENHUIS, S. & MELIEF, C.J.M. (1993). A phase I study of prolonged continuous infusion of low dose recombinant interleukin-2 in melanoma and renal cell cancer. II. Immunological aspects. *Br. J. Cancer*, **68**, 559–567.

WEIL-HILLMAN, G., SCHELL, K., SEGAL, D.M., HANK, J.A., SOSMAN, J.A. & SONDEL, P.M. (1991). Activation of human T cells obtained pre- and post-interleukin-2 (IL-2) therapy by anti-CD3 monoclonal antibody plus IL-2: implications for combined in vivo treatment. *J. Immunother.*, **10**, 267–277.

YOSHINO, I., YANO, T., MURATA, M., ISHIDA, T., SUGIMACHI, K., KIMURA, G. & NOMOTO, K. (1991). Cytolytic potential of peripheral blood T-lymphocytes following adoptive immunotherapy with lymphokin-activated killer cells and low-dose interleukin 2. *Cancer Res.*, **51**, 1494–1498.